# Suspicions of possible vaccine harms must be scrutinised openly and independently to ensure confidence

**DOI:** 10.1038/s41541-020-0202-9

**Published:** 2020-07-06

**Authors:** Karsten Juhl Jørgensen, Margrete Auken, Louise Brinth, Rebecca Chandler, Peter C. Gøtzsche, Tom Jefferson

**Affiliations:** 1 Nordic Cochrane Centre, Rigshospitalet, Dept. 7811, Blegdamsvej 9, 2100 Copenhagen, Denmark; 2 European Parliament, Rue Wiertz 60, 1047 Bruxelles, Belgium; 3 Department of Imaging and Radiology, North Zealand Hospital, University of Copenhagen, Dyrehavevej 29, 3400 Hillerød, Denmark; 4 Research Section, Uppsala Monitoring Centre, Uppsala, Sweden; 5 Honorary Research Fellow, Centre for Evidence Based Medicine, Oxford, OX2 6GG UK

**Keywords:** Drug regulation, Public health

We understand the worry about unfounded vaccine scares raised by Head and colleagues in relation to the debate around HPV vaccines^1^. However, we would like to highlight some broader perspectives, correct some misunderstandings, as well as point to missing information and the possible implications of their criticisms.

First, it is important to make clear that the publications criticised by Head and colleagues^1^ and the European Medicines Agency (EMA)^2^ do not claim a causal link between the HPV vaccines and neurological symptoms^3,4^. The publications by Brinth and colleagues state that their observational data do not provide evidence of causality but are hypothesis generating^3,4^. Our complaint to the EU Ombudsman does not claim that such a link has been established^5^. We complained about what we see as the lack of fairness, reproducibility and transparency of the EMA processes, and we expressed our concerns regarding the handling by EMA of an official report from the Danish Health and Medicines Authority (DHMA) about possible harms of the HPV vaccines and the EMA’s handling of conflicts of interest^5^. This is highlighted on the first page of our complaint^5^.

Head et al. indicate that the report from the DHMA was based solely on the observational studies published by Brinth and colleagues^1^. However, the Uppsala Monitoring Centre (UMC) supported the suspicion that specific neurological symptoms in reports of adverse events after HPV vaccination might be caused by the vaccine^5,6^. Because of the strength of this suspicion, the Danish authorities acted as required and asked the EMA to review the evidence. EMA concluded that there was no convincing evidence of harms^7^.

We do not feel the EMA’s Pharmacovigilance Risk Assessment Committee (PRAC) conducted ‘a detailed and wide-ranging review of the evidence’ as claimed by Head and colleagues^1^. The PRAC asked 5 questions to the ‘market authorisation holders (MAH)’ (i.e. the pharmaceutical companies). The MAHs then performed the evidence review, which included a review of clinical trial data and of passively collected surveillance data. An observed versus expected statistical analysis, based upon spontaneously collected data, was also performed by the MAHs. These formed the basis for the conclusions of the PRAC^2^. We find this practice unsatisfactory, as it is well known that there is a large amount of under-reporting of adverse events into passive surveillance systems^8^.

When the DHMA in 2014 asked Sanofi Pasteur MSD to review its database for potential adverse effects of its HPV vaccine, the head of unit of at the DHMA noted that ‘The company had made some searches and arranged them in such a way that the Danish filed reports were practically not included, and that is no good, as the Danish reports of POTS were well reported and diagnosed at a specialised clinic. Therefore, they needed to be included’^9^. Similar concerns were raised by the DHMA regarding the overall methods and searches used by the MAHs to answer the EMA’s PRAC questions^10^. There is no mention of these concerns in the official EMA report and the search string was not available either^2^. We will share the correspondence between the DHMA and the EMA regarding this upon request.

One of our criticisms was that the search strings used by the MAHs for the EMA report are not available for scrutiny^5^. In addition, the EMA report did not include epidemiological data on the association between HPV vaccines and cases of complex regional pain syndrome (CRPS) and postural orthostatic tachycardia syndrome (POTS) and there was no proposal or recommendation by either the EMA or the MAHs to collect or obtain such data^2,11^.

The review and conclusions of the EMA have not resulted in a renewed confidence in the HPV vaccination programme in Denmark, where vaccine coverage dropped below 25%, down from 90% coverage 5 earlier^12^. This case highlights the general problem of lack of transparency that is caused by allowing pharmaceutical companies to assess whether their products cause adverse effects.

Our complaints to the EMA and to the EU Ombudsman were drafted by the director of the Nordic Cochrane Centre and we therefore used the Centre’s letterhead (which is different from the one used by Cochrane centrally). It is clearly stated on the first page of our complaint that it is from all of us^5^.

We must openly discuss suspected harms of any intervention in healthcare, including vaccines, without comparisons to the Wakefield scandal or ‘anti-vaccine’ groups^1^. If we refute suspicions of vaccine harms without sufficient evidence, we close the door to important improvements in vaccine safety and jeopardise the trustworthiness of our profession. The case of the Pandemrix influenza vaccine has taught us that vaccines may be associated with rare, unexpected, serious neurological harms in certain subpopulations^13–16^. The implications of criticism from colleagues and health authorities against those who raise suspicions about vaccine harms might prevent healthcare professionals from going public or to authorities with well-founded concerns about possible harms in the future, not just about vaccines—which is worrying for healthcare in general.

## References

[1] Head MG, Wind-Mozley M, Flegg PJ (2017). Inadvisable anti-vaccination sentiment: Human Papilloma Virus immunisation falsely under the microscope. npj Vaccines.

[2] European Medicines Agency. *Assessment Report*. *Human Papilloma Virus (HPV) Vaccines*. 40 (European Medicines Agency, 2015).

[3] Brinth LS, Pors K, Theibel AC, Mehlsen J (2015). Orthostatic intolerance and postural tachycardia syndrome as suspected adverse effects of vaccination against human papilloma virus. Vaccine.

[4] Brinth L, Theibel AC, Pors K, Mehlsen J (2015). Suspected side effects to the quadrivalent human papilloma vaccine. Dan. Med J..

[5] Gøtzsche, P. C., Jørgensen, K. J., Jefferson, T., Auken, M. & Brinth, L. Complaint to the European ombudsman over maladministration at the European Medicines Agency (EMA) in relation to the safety of the HPV vaccines. 10 October 2016. http://nordic.cochrane.org/sites/nordic.cochrane.org/files/public/uploads/ResearchHighlights/Complaint-to-ombudsman-over-EMA.pdf (2016).

[6] Chandler RE (2016). Current safety concems with human papillomavirus vaccine: a cluster analysis of reports in Vigibase (R). Drug Saf..

[7] Danish Health Authority. EMA’s new assessment of the HPV vaccine: the benefits outweigh the risks. 17 April 2015. https://www.sst.dk/en/news/2015/emas-new-assessment-of-the-hpv-vaccine-the-benefits-outweigh-the-risks (2015).

[8] Hazell L, Shakir SAW (2006). Under-reporting of adverse drug reactions: a systematic review. Drug Saf..

[9] Weber, C. & Andersen, S. Company behind the HPV-vaccine understated the number of serious side effects. https://www.b.dk/nationalt/firma-bag-hpv-vaccinen-underdrev-omfanget-af-alvorlige-bivirkninger (2015).

[10] European Ombudsman. PRAC (co)-rapporteur’s referral 2nd updated preliminary assessment report. Updated report circulated 28 October 2015. https://www.ombudsman.europa.eu/en/decision/fr/84736 (2015).

[11] Jefferson T, Jørgensen L (2016). Human papilloma virus (HPV) vaccines, complex regional pain syndrome (CRPS), postural orthostatic tachycardia syndrome (POTS), and autonomic dysfunction: a review of the regulatory evidence from the European Medicines Agency (EMA). Indian J. Med. Ethics.

[12] Larsen, K. Danish Health and Medicines Agency prepares vaccine offensive. Ugeskr Laeger. http://ugeskriftet.dk/nyhed/sundhedsstyrelsen-forbereder-vaccine-offensiv (2017).

[13] Sturkenboom MCJM. (2015). The narcolepsy-pandemic influenza story: can the truth ever be unraveled?. Vaccine.

[14] Verstraeten T (2016). Pandemrix TM and narcolepsy: A critical appraisal of the observational studies. Hum. Vaccin Immunother..

[15] Sarkanen TO, Alakuijala APE, Dauvilliers YA, Partinen MM (2018). Incidence of narcolepsy after H1N1 influenza and vaccinations: systematic review and meta-analysis. Sleep. Med Rev..

[16] Feltelius N (2015). A coordinated cross-disciplinary research initiative to address an increased incidence of narcolepsy following the 2009-2010 Pandemrix vaccination programme in Sweden. J. Intern Med.

